# Formulating productive marketing communications strategy: a major health system’s experience

**DOI:** 10.1186/s12913-018-3676-7

**Published:** 2018-12-14

**Authors:** James K. Elrod, John L. Fortenberry

**Affiliations:** 1 Willis-Knighton Health System, 2600 Greenwood Road, Shreveport, LA 71103 USA; 2 LSU Shreveport, 1 University Place, Shreveport, LA 71115 USA

**Keywords:** Marketing communications, Advertising, Promotion, Strategy, Healthcare

## Abstract

**Background:**

Healthcare establishments serve as key community resources, bringing into locales a wealth of resources aimed at enhancing and improving health and wellness. Without effective communications, current and prospective patients will remain unaware of available offerings, foiling opportunities for mutually beneficial exchange. Today, healthcare organizations engage audiences by selecting from among the components of the marketing communications mix, but this wasn’t always the case. There was a time not long ago when communications options were limited due to industry traditions, creating associated challenges.

**Discussion:**

Willis-Knighton Health System faced a communications dilemma in the 1970s when, as a small healthcare provider desirous of growth, it could not achieve a satisfactory media presence via the usual and customary route of the day: submitting press releases to news media organizations, requesting conveyance of associated stories to their audiences. This forced the institution to explore other possibilities, ultimately leading it to experiment with and embrace advertising at a time period when its use was generally shunned in the industry. Willis-Knighton Health System’s pioneering deployment of advertising helped the institution achieve its intended promotions goals, supplying mutual benefits and affording insights which influence its communications approach to this day.

**Conclusions:**

Deploying advertising years in advance of its widespread acceptance and use in the healthcare industry, Willis-Knighton Health System forged new pathways and acquired experience which fostered provider-patient engagement initiatives, affording an enduring marketing communications approach. Challenging situations are quite common in the healthcare industry and the one faced by Willis-Knighton Health System was no exception, but it supplied an immense opportunity to innovate, leading to communications prowess, resulting growth, informed audiences, and lasting mutual benefits.

## Background

Health and medical establishments serve as key community resources, bringing into cities, towns, and other municipalities highly-trained personnel, advanced technologies, and related infrastructure aimed at enhancing and improving the health and wellness of the populace [1–3]. Many, in fact, strive to become true community partners by extending efforts to deliver enhanced value of benefit to the citizenry [1, 4–7]. Recruitment of physicians for the provision of innovative medical services, provision of free health fairs to foster wellness, celebration of new certifications that demonstrate excellence, and so on all constitute noble efforts and achievements worthy of being communicated vigorously. Without effective communications, current and prospective patients will remain unaware of the offerings provided by healthcare entities, foiling opportunities for mutually beneficial exchange. Transactions between provider and patient notably bolster the economic viability of healthcare organizations and, given the nature of offered services, shore up the health and wellness of individuals, making communications excellence imperative for generating productive patronage [8–15].

Today, when progressive healthcare establishments seek to engage patient populations, they generally turn to the marketing communications mix which entails a range of communication categories that can be called upon to connect with customers [11, 16, 17]. The classic array depicted in the marketing communications mix includes advertising (i.e., the paid use of mass media to deliver messages), personal selling (i.e., the use of sales agents to personally deliver messages), sales promotion (i.e., the use of incentives, such as contests and free giveaways, to encourage patronage), direct marketing (i.e., the delivery of messages via mail, the Internet, and similar routes directly to customers), and public relations (i.e., the use of publicity and other unpaid promotional methods to deliver messages) [11, 12]. Healthcare entities select from among these options in a bid to reach target audiences, hoping to encourage them to forward their patronage or engage in some other form of desired exchange [11, 17]. But full use of the marketing communications mix by healthcare providers is a relatively new phenomenon, beginning in the 1980s, decades after its acceptance and use in other industries [8, 10, 18].

Deployment delays of the full marketing communications mix in the healthcare industry resulted from long-standing industry traditions and related practices that limited communications options, making engagement with current and prospective patients especially challenging, particularly for providers who were not in market dominant positions. In the 1970s, Willis-Knighton Health System occupied such a position and, on deciding to pursue a growth and expansion strategy, experienced firsthand the limitations of prevailing industry mindsets regarding communications. With growth and the mutual benefits that it would afford for the institution and its patients at stake, Willis-Knighton Health System decided to forge a new and different communications pathway, with this innovative course fostering its expansion initiatives and providing insights which influence its communications approach to this day.

### Willis-Knighton Health System and its desire to bolster communications

Willis-Knighton Health System is a nongovernmental, not-for-profit healthcare provider delivering comprehensive health and wellness services through multiple hospitals, numerous general and specialty medical clinics, an all-inclusive retirement community, and more. Headquartered in Shreveport, Louisiana, the system holds market leadership in its served region, centered in the heart of an area known as the Ark-La-Tex, where the states of Arkansas, Louisiana, and Texas converge. Willis-Knighton Health System’s origins date to 1924 with the establishment of Tri-State Sanitarium, founded to address the healthcare needs of the burgeoning population of west Shreveport. Sold in 1929 to Drs. James Willis and Joseph Knighton, the establishment continued operations and, in 1952, it was renamed in honor of Drs. Willis and Knighton. Going forward, the institution focused exclusively on serving the population of west Shreveport, but in the 1970s, expansion initiatives beyond this particular geographic area were pursued [19–21]. These initiatives led to dramatic growth in ensuing decades, eventually resulting in comprehensive market coverage and market leadership, acquired in the mid-1990s and continuing to present day [18, 22].

In achieving this lofty market position, Willis-Knighton Health System turned to various structure, product, and process innovations, adopting the hub-and-spoke model of organization design [21, 23], establishing centers of excellence [20], and embracing the practice of adaptive reuse [19, 24], with each of these approaches notably emerging from outside of the healthcare industry [22]. But the institution realized that product-related advancements alone would not be sufficient for achieving its growth objectives; it also had to communicate effectively with target audiences, namely current and prospective patients who, through their patronage, produce all-important customer traffic, setting the stage for the expansion of market share. In this particular era, mass media exposure tended to be acquired via the public relations component of the marketing communications mix whereby healthcare providers would forward press releases profiling noteworthy matters to news media outlets in hopes that they, in turn, would disseminate given stories to their audiences. Advertising was viewed during this period as being beneath the dignity of medical organizations, with some also frowning on the practice due to its potential to upset the time-honored method of patient acquisition: referrals between and among caregivers. The American Medical Association even prohibited its members from engaging in advertising. Other industries had no trepidations concerning the use of advertising and deployed it fully, contrasting significantly with the healthcare industry’s stance [8, 10, 11, 16]. Other elements of the marketing communications mix were used in this era by healthcare entities, however, none of these were delivered via mass media channels, limiting audience exposure.

Willis-Knighton Health System initially pursued the traditional approach of relying on news media outlets to cover its achievements and initiatives, but as a smaller, less prominent healthcare entity, garnering media interest and attention proved to be challenging. Despite the distribution of numerous press releases, many of which sought to publicize free healthcare services for the underprivileged and other altruistic offerings, publication acceptances by news media organizations were few and far between. Willis-Knighton Health System’s experience differed greatly from that enjoyed by the market leader of the day which received frequent and robust coverage of its various pursuits [18]. Within a relatively short experimentation period, it became apparent to executives that telegraphing information via the route traditionally used by healthcare entities would not be fruitful, warranting a revised approach.

### The selection and pursuit of advertising

In seeking a reliable pathway which would permit Willis-Knighton Health System to successfully inform and enlighten audiences, the institution resorted to something that it was becoming increasingly adept at doing: identifying innovations emerging outside of the healthcare industry for use within. This very philosophy had led to numerous product-related advancements which were driving growth and executives believed that it would work equally well when applied to communications. On deciding to pursue this innovative course, it did not take long to identify the obvious pathway: advertising. Age old, heavily studied, highly capable, and extensively used in business and industry, advertising provided a prudent and time-tested method for engaging current and prospective customers. Offered in various formats, including television, radio, newspaper, magazine, billboard, and more, advertising, courtesy of paid placements, provided assurances that messages would be delivered as intended to desired audiences on desired timetables [11, 18, 25–27]. This afforded a major advantage over news media solicitations which were unreliable and, even when coverage was acquired, messages often were heavily edited, diminishing or even distorting supplied content [18].

Although advertising required payment and, at least to some, did not possess the credibility associated with news media coverage, its advantages provided an effective if not superior counterbalance. And while advertising was viewed to be somewhat distasteful by prevailing healthcare industry mindsets of the day, this made little difference, as its pursuit was necessitated by difficulties in securing news media coverage which effectively created a mass communications blackout for the institution [18]. Advertising offered a prudent avenue for reliably and accurately sharing Willis-Knighton Health System’s activities and initiatives, leading to the institution’s associated pursuit. Distribution of press releases to news media organizations was still planned, but by pursuing advertising, the institution no longer had to depend on the decisions of others as to whether or not stories would be communicated.

After investigating options, executives decided to pursue two particular media types: newspaper advertising and billboard advertising. As for newspaper advertising, Willis-Knighton Health System’s initial messages introduced newly recruited physicians, supplying their education and work histories, noting also their contact information. This was a first for the market. It represented an enhancement from that typically provided in news-related accounts stemming from press releases, but used the same mass media channel, reaching the same large audiences. Billboard advertising represented the most novel selection, as the medium had not been used at all by health services organizations in the state of Louisiana. Willis-Knighton Health System became the first, surmising that if billboard advertising worked well for other service-providing entities, such as restaurants and hotels, it would work equally well for healthcare establishments. Its initial billboard advertisement provided a public service message promoting childhood immunizations [13, 18].

### Impact on marketing communications: past, present, future

Willis-Knighton Health System’s initial advertising pursuits were well received by patients, employees, and other publics of the institution and anecdotal evidence strongly suggested the enhancement of awareness, prompting its continued use. On acquiring further experience with equally satisfactory results, the institution embraced advertising fully and, over time, developed an extensive skill set for effectively deploying the medium. This proved highly valuable as, in the 1980s, resistance against health services advertising faltered, helped by the US Federal Trade Commission’s scrutiny of the American Medical Association’s ban on its members’ use of advertising which subsequently was relinquished [8]. As such, Willis-Knighton Health System had an advantage over entities which were just beginning to explore the medium.

These formative experiences paved the way for the establishment of formal marketing operations well in advance of their proliferation in the industry, extending competitive advantages which, in part, are credited for the institution’s eventual emergence as market leader [18]. They also fostered the development of three institutional perspectives which influence Willis-Knighton Health System’s communications approach to this day; namely that (1) communications excellence is mandatory, as institutional prosperity and the health and wellness of those served depend on productively engaging audiences, (2) communications innovations should be explored across all industries for potential use, with experimentation being encouraged, something which openly discourages insular mindsets that can limit healthcare industry advancements, and (3) given the importance of communicating with audiences, core methods must be reliable, with conveyances being assured, as in the case of advertising, rather than hoped for, as in the case of news media solicitations.

Today, Willis-Knighton Health System actively pursues the full range of communications options in the marketing communications mix. Advertising constitutes Willis-Knighton Health System’s most used promotional method, with numerous ad media platforms (e.g., television, newspaper, magazine, billboard) being called upon to convey various messages. Personal selling is used through the employment of community liaisons who essentially serve as sales representatives, representing the institution in the marketplace and educating audiences regarding available services and support. Direct marketing is called upon through the use of direct mail whereby promotional parcels are forwarded to individuals via the US Postal Service, and increasingly, the Internet through email and social media campaigns. Sales promotion is used more modestly, usually through the distribution of free items, such as logo-bearing pens, calendars, and similar objects, to patients and guests. Public relations activities are numerous, including open houses, informative seminars, health fairs, and the like, but Willis-Knighton Health System’s plan for conveying such via mass media remains the same as it was decades earlier: prepare and submit press releases to local news media organizations, but rely on advertising for informing audiences of the associated events and opportunities. Notably, even after acquiring market leadership, the number of publication acceptances and, of those accepted, the quality of presentation, remains unsatisfactory, reaffirming the institution’s position on advertising reliance.

As for future treatment of the marketing communications mix, Willis-Knighton Health System plans to continue its present approach, shifting allocations as needed to ensure excellent connectivity with patient populations, with an eye always focused externally in search of innovative methods that might be used within. The most exciting communications development of late pertains to the emergence of new media pathways which use the Internet to engage audiences. Social media websites, such as Facebook, Twitter, and Instagram, offer significant opportunities for establishing dialogues with customers and society [28–30]. Essentially a form of direct marketing, Willis-Knighton Health System expects proficiencies in this area to become all the more important in coming years and it is actively working to develop associated platforms and skills. The digital revolution also has impacted advertising due to the extensive and expansive use of smartphones and other electronic devices by consumers [31]. As such, digital advertising is consuming an increasing share of the institution’s attention and marketing communications budget, with print advertising retracting somewhat as the technological age continues to evolve and alter the information consumption preferences of audiences. Courtesy of new and anticipated technological advancements, opportunities to more richly engage customers indeed abound.

## Conclusions

There is no substitute for experience and Willis-Knighton Health System received just that, serving by circumstance and situation as a pioneer in health services advertising during a very interesting time period in the healthcare industry. Deploying the medium years in advance of its widespread acceptance and use in health services organizations, the pathways forged and experience acquired helped the institution achieve its intended conveyance and engagement goals of the era, affording a marketing communications approach that remains fruitful. With the proliferation of healthcare advertising being so pronounced in present day, a time when such conveyances were limited and even restricted by tradition and practice now seems unimaginable. Ultimately, the free circulation of information and the benefits afforded by such prevailed, changing the course of industry communications. Challenging situations are quite common in the healthcare industry and the one faced by Willis-Knighton Health System was no exception, but it supplied an immense opportunity to innovate, leading to communications prowess, resulting growth, informed audiences, and lasting mutual benefits.
